# (5,7-Dimethyl-2-oxo-2*H*-chromen-4-yl)methyl diethyl­dithio­carbamate

**DOI:** 10.1107/S1600536812019757

**Published:** 2012-05-05

**Authors:** K. Mahesh Kumar, H. C. Devarajegowda, S Jeyaseelan, N. M. Mahabaleshwaraiah, O. Kotresh

**Affiliations:** a Department of Chemistry, Karnatak Science College, Dharwad 580 001, Karnataka, India; b Department of Physics, Yuvaraja’s College (Constituent College), University of Mysore, Mysore 570 005, Karnataka, India

## Abstract

In the title compound, C_17_H_21_NO_2_S_2_, the coumarin ring system is nearly planar, with a maximum deviation of 0.080 (2) Å from the mean plane. An intra­molecular C—H⋯S hydrogen bond occurs. The crystal structure features C—H⋯S hydrogen bonds and weak π–π inter­actions with a centroid–centroid distance of 3.679 (1) Å.

## Related literature

For biological applications of coumarins and dithio­carbamates, see: Smith *et al.* (1998 ▶); Nawrot-Modraka *et al.* (2006 ▶); Basanagouda *et al.* (2009 ▶); Kalkhambkar *et al.* (2007 ▶); El-Shorbagi (2000 ▶); Ronconi *et al.* (2006 ▶); Cvek & Dvorak (2007 ▶). For a related structure, see: Kumar *et al.* (2012 ▶). For the synthesis of the title compound, see: Shastri *et al.* (2004 ▶).
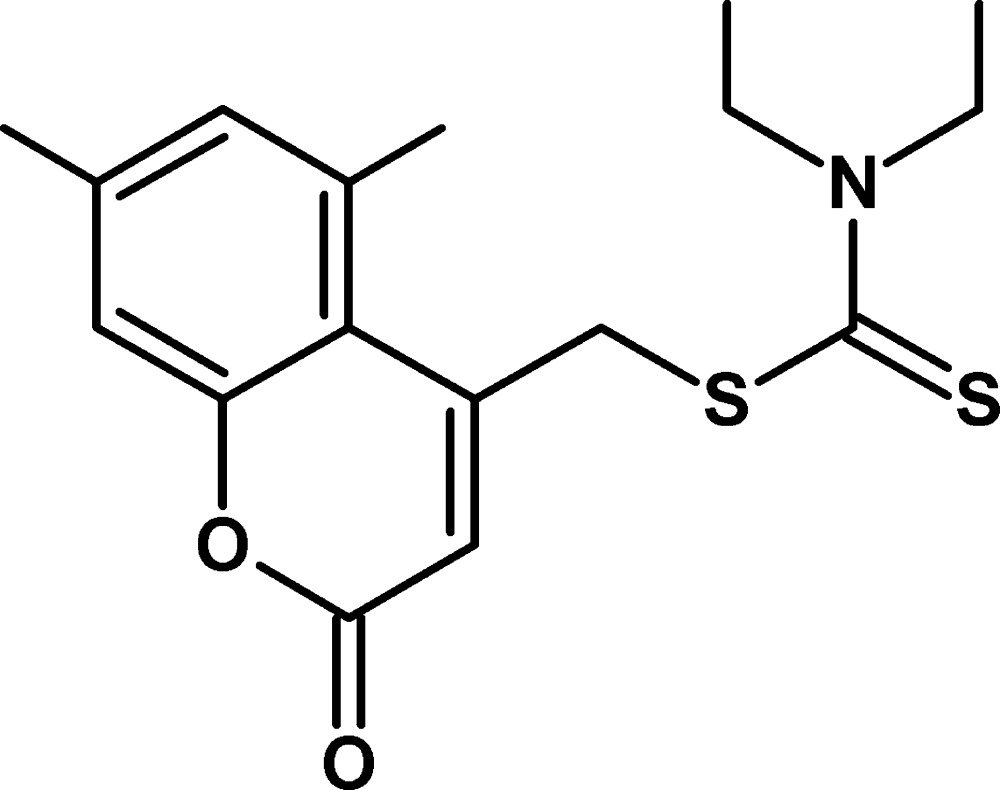


## Experimental

### 

#### Crystal data

C_17_H_21_NO_2_S_2_
*M* *_r_* = 335.47
Monoclinic, 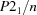

*a* = 7.8570 (2) Å
*b* = 23.7745 (5) Å
*c* = 9.7684 (2) Å
β = 109.483 (1)°
*V* = 1720.22 (7) Å^3^
*Z* = 4
Mo *K*α radiation
μ = 0.32 mm^−1^
*T* = 293 K
0.24 × 0.20 × 0.12 mm

#### Data collection

Bruker SMART CCD area-detector diffractometer
Absorption correction: ψ scan (*SADABS*; Sheldrick, 2007 ▶) *T*_min_ = 0.770, *T*_max_ = 1.000
14939 measured reflections
3033 independent reflections
2803 reflections with *I* > 2σ(*I*)
*R*_int_ = 0.020

#### Refinement

*R*[*F* ^2^ > 2σ(*F* ^2^)] = 0.051
*wR*(*F* ^2^) = 0.135
*S* = 1.04
3033 reflections
204 parameters
H-atom parameters constrained
Δρ_max_ = 0.90 e Å^−3^
Δρ_min_ = −0.73 e Å^−3^

### 

Data collection: *SMART* (Bruker, 2001 ▶); cell refinement: *SAINT* (Bruker, 2001 ▶); data reduction: *SAINT*; program(s) used to solve structure: *SHELXS97* (Sheldrick, 2008 ▶); program(s) used to refine structure: *SHELXL97* (Sheldrick, 2008 ▶); molecular graphics: *ORTEP-3* (Farrugia, 1997 ▶); software used to prepare material for publication: *SHELXL97*.

## Supplementary Material

Crystal structure: contains datablock(s) I, global. DOI: 10.1107/S1600536812019757/wn2474sup1.cif

Structure factors: contains datablock(s) I. DOI: 10.1107/S1600536812019757/wn2474Isup2.hkl

Supplementary material file. DOI: 10.1107/S1600536812019757/wn2474Isup3.cml

Additional supplementary materials:  crystallographic information; 3D view; checkCIF report

## Figures and Tables

**Table 1:** Hydrogen-bond geometry (Å, °)

*D*—H⋯*A*	*D*—H	H⋯*A*	*D*⋯*A*	*D*—H⋯*A*
C8—H8⋯S2^i^	0.93	2.86	3.751 (2)	161
C16—H16*C*⋯S1	0.96	2.53	3.282 (3)	135

Symmetry code: (i) 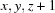.

**Fig. 1.**
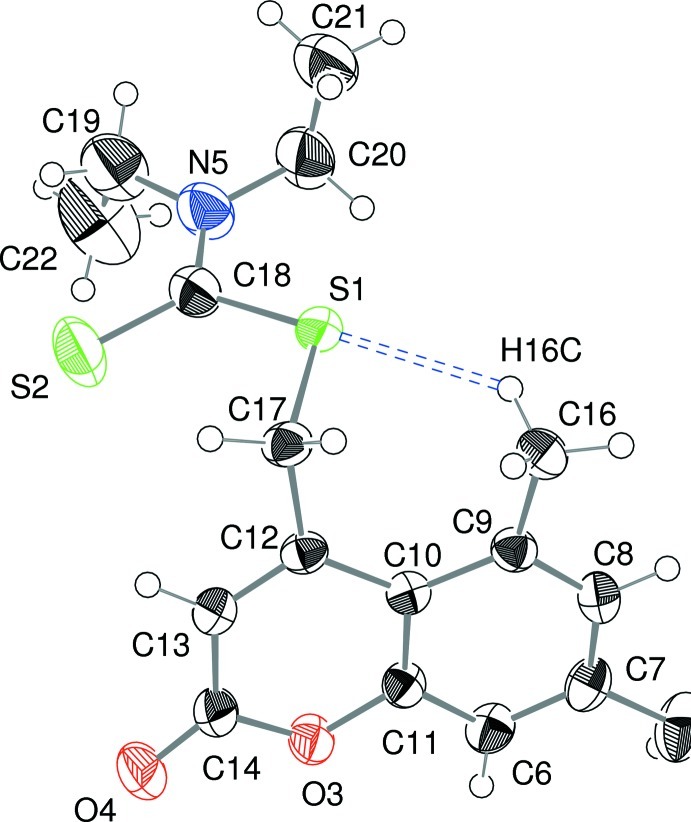
The molecular structure of the title compound. Displacement ellipsoids are drawn at the 50% probability level. Hydrogen atoms are shown as spheres of arbitrary radius. The dashed line indicates the intramolecular hydrogen bond.

**Fig. 2.**
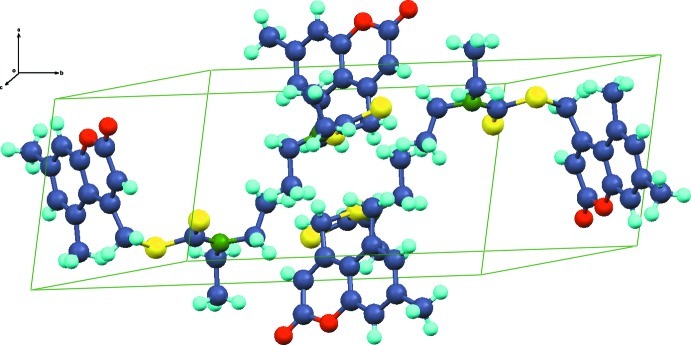
The packing of the molecules in the crystal structure.

## supplementary crystallographic information

### Comment

Coumarins constitute a class of compounds which are found widely in nature and possess diverse biological activities. Over recent decades, medicinal chemists have paid great attention to the isolation, screening and structural modifications of new coumarins. They have been found to exhibit a wide range of applications in cancer, the HIV drug development arena (Smith *et al.,* 1998), anti-tumor, anti-bacterial and cytotoxic activity (Nawrot-Modraka *et al.,* 2006). In 4-substituted coumarins, the groups attached at the C-4 methylene carbon been shown to influence their solid state conformations, as observed in 4-aryloxymethyl (Basanagouda *et al.,* 2009) and 4-arylaminomethyl coumarins (Kalkhambkar *et al.,* 2007).

Dithiocarbamates have shown wide applications as pesticides,fungicides in agriculture (El-Shorbagi, 2000), potent anticancer agents (Ronconi *et al.,* 2006), organic intermediates, rubber additives, additives of polluted water and vulcanizing agents (Cvek & Dvorak, 2007).

In view of the above observations,we proposed that 4-substituted coumarins bearing the dithiocarbamate (DTC)group should display some interesting biological activity and the title compound was screened for fungicidal, bacterial and DNA cleavage properties.The crystal structure of a coumarin derivative linked to the DTC group has been reported (Kumar *et al.,* 2012).

The title compound is one of a series of dithiocarbamate coumarins with potential as possible anti-microbial agents. For these reasons, in continuation of our interest in the crystal structures of coumarin derivatives, we report here its crystal structure.

The asymmetric unit of 5,7-dimethyl-2-oxo-2*H*-chromen-4-yl)methyl diethyldithiocarbamate is shown in Fig. 1.The coumarin ring system (O3/C6–C14) is nearly planar, with a maximum deviation from the mean plane of 0.080 (2) Å for atom C12.

In the crystal structure (Fig. 2), the molecules are connected *via* weak intramolecular C8—H8···S2 and intermolecular C16—H16C···S1 hydrogen bonds (Table 1). Furthermore, the crystal structure features π-π stacking interactions between the pyran ring (O3/C10–C14; centroid *Cg1*) and the benzene ring (C6–C11; centroid *Cg2*), with a *Cg1*···*Cg2* distance of 3.679 (1) Å.

### Experimental

All the chemicals were of analytical reagent grade and were used directly without further purification. 4-Bromomethyl coumarin required for the synthesis of the target molecule was synthesized according to an already reported procedure involving Pechmann cyclization of phenols with 4-bromoethyl acetoacetate (Shastri *et al.,* 2004) and sodium diethyldithiocarbamate purchased from Sigma- Aldrich.

A mixture of 2.6 g (0.01 mol) of 5,7-dimethyl-4-bromomethylcoumarin and 1.71 g (0.01 mol) of sodium diethyldithiocarbamate in 30 ml dry alcohol was stirred for 24 h at room temperature (the reaction was monitored by TLC). The solvent was evaporated and the resulting solid was extracted twice with a dichloromethane-H_2_O mixture. The organic layer was dried over anhydrous CaCl_2_ and evaporation of the organic solvent gave the title compound. The compound was recrystallized from an ethanol-chloroform mixture. Colour: Colourless. Yield: 91%. M.P.: 409 K.

### Refinement

All H atoms were positioned geometrically [Csp^2^—H = 0.93 Å, C(methylene)—H = 0.97 Å and C(methyl)—H = 0.96 Å] and refined using a riding model with *U*_iso_(H) = 1.5*U*_eq_(C) for methyl H and *U*_iso_(H) = 1.2*U*_eq_(C) for other H.

### Crystal data

**Table:**

C_17_H_21_NO_2_S_2_	*F*(000) = 712
*M**_r_* = 335.47	*D*_x_ = 1.295 Mg m^−^^3^
Monoclinic, *P*2_1_/*n*	Melting point: 409 K
Hall symbol: -P 2yn	Mo *K*α radiation, λ = 0.71073 Å
*a* = 7.8570 (2) Å	Cell parameters from 3033 reflections
*b* = 23.7745 (5) Å	θ = 1.7–25.0°
*c* = 9.7684 (2) Å	µ = 0.32 mm^−^^1^
β = 109.483 (1)°	*T* = 293 K
*V* = 1720.22 (7) Å^3^	Plate, colourless
*Z* = 4	0.24 × 0.20 × 0.12 mm

### Data collection

**Table:**

Bruker SMART CCD area-detector diffractometer	3033 independent reflections
Radiation source: fine-focus sealed tube	2803 reflections with *I* > 2σ(*I*)
Graphite monochromator	*R*_int_ = 0.020
ω and φ scans	θ_max_ = 25.0°, θ_min_ = 1.7°
Absorption correction: ψ scan (*SADABS*; Sheldrick, 2007)	*h* = −9→9
*T*_min_ = 0.770, *T*_max_ = 1.000	*k* = −26→28
14939 measured reflections	*l* = −11→11

### Refinement

**Table:**

Refinement on *F*^2^	Secondary atom site location: difference Fourier map
Least-squares matrix: full	Hydrogen site location: inferred from neighbouring sites
*R*[*F*^2^ > 2σ(*F*^2^)] = 0.051	H-atom parameters constrained
*wR*(*F*^2^) = 0.135	*w* = 1/[σ^2^(*F*_o_^2^) + (0.0643*P*)^2^ + 1.5942*P*] where *P* = (*F*_o_^2^ + 2*F*_c_^2^)/3
*S* = 1.04	(Δ/σ)_max_ = 0.003
3033 reflections	Δρ_max_ = 0.90 e Å^−^^3^
204 parameters	Δρ_min_ = −0.73 e Å^−^^3^
0 restraints	Extinction correction: *SHELXL97* (Sheldrick, 2008), Fc^*^=kFc[1+0.001xFc^2^λ^3^/sin(2θ)]^-1/4^
Primary atom site location: structure-invariant direct methods	Extinction coefficient: 0.0052 (14)

### Special details

**Table:**

Experimental. IR (KBr) 670 c m^-1^ (C—S), 1204 c m^-1^ (C=S), 1047 c m^-1^ (C—O), 823 c m^-1^ (C—N),1279 c m^-1^ (C—O—C), 1716 c m^-1^ (C=O).GCMS data m/e = 335. 1H NMR (400 MHz, CDCl_3_, δ, p.p.m.): 1.58 (d Ethylene-6H, CH_3_), 2.46 (s,3*H*, CH_3_), 2.71 (s,3*H*, CH_3_), 3.88 (s,2*H*, Ethylene-CH_2_), 4.21 (s,2*H*, Ethylene-CH_2_), 4.50 (s 2H, Methylene-CH_2_), 6.53 (s,1*H*, Ar—H), 6.92 (s,1*H*, Ar—H), 7.03 (s,1*H*, Ar—H).
Geometry. All s.u.'s (except the s.u. in the dihedral angle between two l.s. planes) are estimated using the full covariance matrix. The cell s.u.'s are taken into account individually in the estimation of s.u.'s in distances, angles and torsion angles; correlations between s.u.'s in cell parameters are only used when they are defined by crystal symmetry. An approximate (isotropic) treatment of cell s.u.'s is used for estimating s.u.'s involving l.s. planes.
Refinement. Refinement of *F*^2^ against ALL reflections. The weighted *R*-factor *wR* and goodness of fit *S* are based on *F*^2^, conventional *R*-factors *R* are based on *F*, with *F* set to zero for negative *F*^2^. The threshold expression of *F*^2^ > 2σ(*F*^2^) is used only for calculating *R*-factors(gt) *etc*. and is not relevant to the choice of reflections for refinement. *R*-factors based on *F*^2^ are statistically about twice as large as those based on *F*, and *R*- factors based on ALL data will be even larger.

### Fractional atomic coordinates and isotropic or equivalent isotropic displacement parameters (Å^2^)

**Table:**

	*x*	*y*	*z*	*U*_iso_*/*U*_eq_	
S1	0.15831 (9)	0.15127 (3)	0.66095 (6)	0.0434 (2)	
S2	0.25909 (15)	0.14818 (4)	0.38884 (10)	0.0773 (3)	
O3	0.77521 (19)	0.04484 (8)	0.88072 (17)	0.0414 (4)	
O4	0.8065 (3)	0.01657 (10)	0.6765 (2)	0.0610 (6)	
N5	0.1516 (4)	0.23806 (10)	0.4975 (3)	0.0583 (6)	
C6	0.7641 (3)	0.06546 (11)	1.1089 (2)	0.0395 (5)	
H6	0.8887	0.0606	1.1437	0.047*	
C7	0.6727 (3)	0.07803 (10)	1.2025 (2)	0.0401 (5)	
C8	0.4863 (3)	0.08315 (10)	1.1453 (2)	0.0378 (5)	
H8	0.4236	0.0902	1.2091	0.045*	
C9	0.3886 (3)	0.07847 (9)	0.9997 (2)	0.0329 (5)	
C10	0.4826 (3)	0.06808 (9)	0.9000 (2)	0.0294 (5)	
C11	0.6692 (3)	0.06000 (9)	0.9621 (2)	0.0327 (5)	
C12	0.4095 (3)	0.06440 (9)	0.7418 (2)	0.0312 (5)	
C13	0.5170 (3)	0.04836 (10)	0.6673 (2)	0.0380 (5)	
H13	0.4675	0.0459	0.5668	0.046*	
C14	0.7056 (3)	0.03479 (11)	0.7350 (3)	0.0405 (5)	
C15	0.7704 (4)	0.08551 (15)	1.3626 (3)	0.0628 (8)	
H15A	0.7936	0.0493	1.4088	0.094*	
H15B	0.6971	0.1074	1.4042	0.094*	
H15C	0.8827	0.1046	1.3770	0.094*	
C16	0.1861 (3)	0.08263 (12)	0.9590 (3)	0.0465 (6)	
H16A	0.1543	0.0847	1.0456	0.070*	
H16B	0.1310	0.0500	0.9041	0.070*	
H16C	0.1442	0.1158	0.9016	0.070*	
C17	0.2169 (3)	0.07796 (10)	0.6516 (2)	0.0369 (5)	
H17A	0.1363	0.0548	0.6844	0.044*	
H17B	0.1982	0.0683	0.5512	0.044*	
C18	0.1913 (4)	0.18349 (11)	0.5078 (3)	0.0480 (6)	
C19	0.1622 (6)	0.27172 (15)	0.3738 (4)	0.0795 (11)	
H19A	0.1363	0.2478	0.2887	0.095*	
H19B	0.0718	0.3012	0.3524	0.095*	
C20	0.0865 (5)	0.26865 (13)	0.6014 (4)	0.0666 (9)	
H20A	0.1272	0.3074	0.6075	0.080*	
H20B	0.1384	0.2519	0.6968	0.080*	
C21	−0.1163 (5)	0.26762 (16)	0.5582 (4)	0.0793 (10)	
H21A	−0.1682	0.2849	0.4647	0.119*	
H21B	−0.1529	0.2879	0.6286	0.119*	
H21C	−0.1570	0.2294	0.5538	0.119*	
C22	0.3417 (7)	0.29714 (19)	0.4049 (7)	0.1145 (17)	
H22A	0.3700	0.3194	0.4917	0.172*	
H22B	0.3420	0.3206	0.3251	0.172*	
H22C	0.4303	0.2680	0.4184	0.172*	

### Atomic displacement parameters (Å^2^)

**Table:**

	*U*^11^	*U*^22^	*U*^33^	*U*^12^	*U*^13^	*U*^23^
S1	0.0513 (4)	0.0436 (4)	0.0340 (3)	0.0154 (3)	0.0127 (3)	0.0011 (2)
S2	0.1254 (8)	0.0562 (5)	0.0777 (6)	0.0240 (5)	0.0703 (6)	0.0081 (4)
O3	0.0260 (8)	0.0627 (11)	0.0369 (9)	0.0014 (7)	0.0124 (6)	0.0009 (8)
O4	0.0491 (11)	0.0906 (16)	0.0508 (11)	0.0181 (10)	0.0267 (9)	−0.0012 (10)
N5	0.0738 (16)	0.0432 (13)	0.0683 (16)	0.0112 (11)	0.0373 (13)	0.0056 (11)
C6	0.0270 (11)	0.0512 (14)	0.0360 (12)	−0.0054 (10)	0.0046 (9)	0.0016 (10)
C7	0.0441 (13)	0.0425 (13)	0.0300 (11)	−0.0084 (10)	0.0074 (10)	−0.0005 (9)
C8	0.0424 (13)	0.0403 (13)	0.0351 (12)	−0.0017 (10)	0.0188 (10)	−0.0017 (9)
C9	0.0311 (11)	0.0325 (11)	0.0361 (12)	0.0004 (9)	0.0126 (9)	0.0013 (9)
C10	0.0270 (10)	0.0287 (10)	0.0318 (11)	−0.0028 (8)	0.0090 (9)	0.0004 (8)
C11	0.0279 (11)	0.0383 (12)	0.0331 (11)	−0.0031 (9)	0.0117 (9)	0.0024 (9)
C12	0.0303 (11)	0.0283 (10)	0.0320 (11)	−0.0002 (8)	0.0065 (9)	0.0002 (8)
C13	0.0389 (12)	0.0439 (13)	0.0294 (11)	0.0050 (10)	0.0091 (9)	0.0002 (9)
C14	0.0389 (12)	0.0498 (14)	0.0366 (12)	0.0040 (11)	0.0177 (10)	0.0036 (10)
C15	0.0609 (18)	0.087 (2)	0.0334 (14)	−0.0135 (16)	0.0061 (12)	−0.0067 (14)
C16	0.0348 (13)	0.0618 (16)	0.0475 (14)	0.0073 (11)	0.0199 (11)	0.0059 (12)
C17	0.0332 (12)	0.0381 (12)	0.0338 (12)	0.0033 (9)	0.0035 (9)	−0.0026 (9)
C18	0.0528 (15)	0.0451 (14)	0.0500 (15)	0.0078 (11)	0.0224 (12)	0.0020 (11)
C19	0.104 (3)	0.0531 (18)	0.101 (3)	0.0124 (18)	0.060 (2)	0.0154 (18)
C20	0.097 (2)	0.0421 (15)	0.069 (2)	0.0145 (15)	0.0382 (18)	−0.0025 (14)
C21	0.094 (3)	0.072 (2)	0.086 (2)	0.0324 (19)	0.049 (2)	0.0158 (19)
C22	0.132 (4)	0.079 (3)	0.165 (5)	−0.026 (3)	0.093 (4)	−0.017 (3)

### Geometric parameters (Å, º)

**Table:**

S1—C18	1.775 (3)	C13—C14	1.443 (3)
S1—C17	1.813 (2)	C13—H13	0.9300
S2—C18	1.659 (3)	C15—H15A	0.9600
O3—C14	1.365 (3)	C15—H15B	0.9600
O3—C11	1.377 (3)	C15—H15C	0.9600
O4—C14	1.202 (3)	C16—H16A	0.9600
N5—C18	1.330 (4)	C16—H16B	0.9600
N5—C20	1.472 (4)	C16—H16C	0.9600
N5—C19	1.474 (4)	C17—H17A	0.9700
C6—C7	1.371 (3)	C17—H17B	0.9700
C6—C11	1.384 (3)	C19—C22	1.469 (6)
C6—H6	0.9300	C19—H19A	0.9700
C7—C8	1.388 (3)	C19—H19B	0.9700
C7—C15	1.505 (3)	C20—C21	1.506 (5)
C8—C9	1.377 (3)	C20—H20A	0.9700
C8—H8	0.9300	C20—H20B	0.9700
C9—C10	1.426 (3)	C21—H21A	0.9600
C9—C16	1.510 (3)	C21—H21B	0.9600
C10—C11	1.400 (3)	C21—H21C	0.9600
C10—C12	1.461 (3)	C22—H22A	0.9600
C12—C13	1.341 (3)	C22—H22B	0.9600
C12—C17	1.510 (3)	C22—H22C	0.9600
			
C18—S1—C17	105.17 (12)	C9—C16—H16B	109.5
C14—O3—C11	122.57 (17)	H16A—C16—H16B	109.5
C18—N5—C20	123.8 (2)	C9—C16—H16C	109.5
C18—N5—C19	121.0 (2)	H16A—C16—H16C	109.5
C20—N5—C19	115.1 (2)	H16B—C16—H16C	109.5
C7—C6—C11	119.4 (2)	C12—C17—S1	113.43 (15)
C7—C6—H6	120.3	C12—C17—H17A	108.9
C11—C6—H6	120.3	S1—C17—H17A	108.9
C6—C7—C8	117.8 (2)	C12—C17—H17B	108.9
C6—C7—C15	121.3 (2)	S1—C17—H17B	108.9
C8—C7—C15	120.8 (2)	H17A—C17—H17B	107.7
C9—C8—C7	124.0 (2)	N5—C18—S2	124.2 (2)
C9—C8—H8	118.0	N5—C18—S1	112.87 (19)
C7—C8—H8	118.0	S2—C18—S1	122.90 (16)
C8—C9—C10	118.8 (2)	C22—C19—N5	111.5 (4)
C8—C9—C16	116.2 (2)	C22—C19—H19A	109.3
C10—C9—C16	124.9 (2)	N5—C19—H19A	109.3
C11—C10—C9	115.73 (19)	C22—C19—H19B	109.3
C11—C10—C12	115.79 (19)	N5—C19—H19B	109.3
C9—C10—C12	128.48 (19)	H19A—C19—H19B	108.0
O3—C11—C6	113.68 (19)	N5—C20—C21	112.2 (3)
O3—C11—C10	122.26 (19)	N5—C20—H20A	109.2
C6—C11—C10	124.0 (2)	C21—C20—H20A	109.2
C13—C12—C10	119.62 (19)	N5—C20—H20B	109.2
C13—C12—C17	115.74 (19)	C21—C20—H20B	109.2
C10—C12—C17	124.64 (19)	H20A—C20—H20B	107.9
C12—C13—C14	123.5 (2)	C20—C21—H21A	109.5
C12—C13—H13	118.3	C20—C21—H21B	109.5
C14—C13—H13	118.3	H21A—C21—H21B	109.5
O4—C14—O3	117.4 (2)	C20—C21—H21C	109.5
O4—C14—C13	127.0 (2)	H21A—C21—H21C	109.5
O3—C14—C13	115.58 (19)	H21B—C21—H21C	109.5
C7—C15—H15A	109.5	C19—C22—H22A	109.5
C7—C15—H15B	109.5	C19—C22—H22B	109.5
H15A—C15—H15B	109.5	H22A—C22—H22B	109.5
C7—C15—H15C	109.5	C19—C22—H22C	109.5
H15A—C15—H15C	109.5	H22A—C22—H22C	109.5
H15B—C15—H15C	109.5	H22B—C22—H22C	109.5
C9—C16—H16A	109.5		

### Hydrogen-bond geometry (Å, º)

**Table:**

*D*—H···*A*	*D*—H	H···*A*	*D*···*A*	*D*—H···*A*
C8—H8···S2^i^	0.93	2.86	3.751 (2)	161
C16—H16*C*···S1	0.96	2.53	3.282 (3)	135

Symmetry code: (i) *x*, *y*, *z*+1.
